# Nonnegative matrix factorization with Wasserstein metric-based regularization for enhanced text embedding

**DOI:** 10.1371/journal.pone.0314762

**Published:** 2024-12-05

**Authors:** Mingming Li, Xingjie Wang, Chunhua Li, Anping Zeng

**Affiliations:** School of Computer Science and Technology, Yibin University, Yibin, Sichuan, China; Ningbo University of Technology, CHINA

## Abstract

Text embedding plays a crucial role in natural language processing (NLP). Among various approaches, nonnegative matrix factorization (NMF) is an effective method for this purpose. However, the standard NMF approach, fundamentally based on the bag-of-words model, fails to utilize the contextual information of documents and may result in a significant loss of semantics. To address this issue, we propose a new NMF scheme incorporating a regularization term based on the Wasserstein metric, which quantifies an approximation of a word-context matrix to leverage semantic information. Since the word-context matrix is typically symmetric and positive definite (SPD), the Wasserstein metric used in the regularization is related to a natural SPD manifold structure. We then build upon this manifold structure to integrate an explicit expression of the Wasserstein metric into the NMF framework. Additionally, we introduce modifications to the gradient computation to adapt three fundamental classes of numerical algorithms from ordinary NMF to tackle the Wasserstein-regularized NMF (NMF-WR) problem. To demonstrate the effectiveness of the NMF-WR model and the proposed algorithms, we conduct experiments on popular datasets, focusing on topic modeling and document clustering. The results indicate that the NMF-WR model exhibits superior performance compared to other conventional NMF models in processing text embeddings. These findings suggest that this novel NMF-WR framework not only enhances semantic representation but also underscores a commitment to the model’s reliability and interpretability.

## Introduction

Text embedding, a pivotal technique in natural language processing (NLP), has contributed significantly to the development of various text processing tasks, such as information retrieval, document classification, document clustering, and text generation, among others [1–3]. With the extensive use of sophisticated neural network models in the field of NLP research, it becomes crucial to represent different levels of textual information—such as words, sentences, or documents—as appropriate mathematical objects embedded within a latent space. These objects should be designed to feed seamlessly into complex neural network models.

The bag-of-words model is among the simplest and most commonly used text representations; however, there are two major drawbacks to this simple model. One issue is the potential for a dimensionality problem, as the representation is typically high-dimensional. The other is that the model usually neglects the word order and contextual information within documents. Based on the bag-of-words model, documents are typically represented by (possibly weighted) term frequency vectors, which can then be compiled into a term-document matrix for a collection of documents [4].

One class of approaches to providing an alternative to the ordinary vector space representation results from the factorization of the term-document matrix. Two specialized examples of this are the singular value decomposition (SVD) [5] and the nonnegative matrix factorization (NMF) [6]. While the SVD has been successful in employing models based on latent semantic analysis (LSA) in extensive text mining and indexing tasks for large datasets [7, 8], its further application is sometimes limited due to the lack of intuitive interpretation. In order to preserve the nonnegativity of the original term-document matrix and to produce interpretable matrix factors, NMF has been introduced as an effective approach in the context of text retrieval. To approximate the original term-document matrix, NMF decomposes it into the product of two reduced-rank, nonnegative matrix factors. These two factors contain the low-dimensional representations of terms and documents, respectively, which are suitable for use as interpretable features across the document corpus. It turns out that both the term factor and the document factor are encoded into a common latent semantic space determined by NMF, making it possible to implement word and document embedding simultaneously. In brief, NMF utilizes mathematical factorization to explore the implicit semantics of natural language text and provides an automatic method for processing text that is robust enough to deal with large-scale information.

As the volume of text for analysis continues to grow, NMF has proven to be well suited to many applications, particularly in terms of document clustering and classification [9]. Furthermore, one of the significant benefits of using NMF for these applications is that the dimensionality problem of the bag-of-words model can be effectively circumvented. This is achieved because the low-rank approximation (i.e., approximating a matrix by one whose rank is less than that of the original matrix) is commonly employed in the NMF framework. However, the absence of contextual information remains a problem due to the neglect of word order in the term-document matrix representation. This is a crucial issue, as it may lead to a significant loss of information regarding semantic relations between words. For example, synonyms cannot be guaranteed to be embedded in the same direction within the latent semantic space.

Unlike LSA and NMF methods, neural network-based methods utilize contextual information to construct embedding models without the need to specify additional structural restrictions. These methods are typically built from large-scale corpora of documents. Two widely used examples are Word2Vec [10] and GloVe [11]. These approaches enable efficient training and evaluation of text data, resulting in useful word embeddings. To date, no other pretrained word embedding models have been found to significantly outperform them. Apart from their effectiveness, these methods also have the advantage of being applicable to complex neural networks [12, 13]. Some of these networks are used for document classification, such as recurrent neural networks [14]. However, neural methods for text embedding may still face several challenges, including a lack of interpretability and computational complexity. To address these issues, alternative schemes for text embeddings have been proposed in the literature, such as probability-based embeddings and manifold-based embeddings.

In recent years, there has been a growing popularity of contextualized embeddings, where word representations may vary with local contexts. Among these, state-of-the-art models include BERT [15], GPT [16], RoBERTa [17], and others. Whereas global embeddings like Word2vec and GloVe, where words have fixed representations across a given corpus, still attract theoretical investigations due to their effectiveness in applications. It has been shown that the skip-gram model with negative sampling (SGNS) within the Word2vec framework can be mathematically explained as implicitly factorizing a Pointwise Mutual Information (PMI) word-context matrix [18]. However, despite the resemblance of PMI matrix factorization to well-established factorization techniques such as SVD and NMF, little attention has been paid to constructing a framework that combines these factorization techniques. Overall, although new research on matrix approximation [19–21] continues to emerge, there is still a need for a modified NMF model that can utilize both the word-context matrix and the term-document matrix.

In this paper, we aim to incorporate word-context matrix factorization into the Non-negative Matrix Factorization (NMF) framework to address the issue of semantic loss. To achieve this, we propose a new NMF model by adding a regularization term to the mathematical formulation of the cost function. Existing regularization approaches for NMF primarily rely on the shrinkage of the resulting factor matrices by imposing a penalty on their numerical magnitude; however, these methods still result in the loss of semantic information. Our proposed regularization term is inspired by quantifying the approximation due to the word-context matrix factorization. A key aspect of implementing this strategy is the selection of an appropriate metric for evaluation. Given that the word-context matrix is symmetric and positive definite (SPD), we consider the Wasserstein metric, which captures the unique manifold structure of the space of SPD matrices.

Indeed, there is extensive research on Wasserstein embeddings-based NMF models in the literature [22]. However, these models are limited to using Wasserstein embeddings to factorize the term-document matrix, and none of them are constructed within the SPD manifold framework. In our work, the proposed Wasserstein metric is specifically designed to handle the factorization of the word-context matrix and is related to the aforementioned SPD manifold structure, which has been shown to have a natural geometric interpretation [23]. This structure is thus promising for use in specially designed algorithms for practical applications such as topic modeling and document clustering.

For the purposes of this paper, we adapt three basic algorithms—multiplicative update, gradient descent, and alternating least squares—from the standard NMF framework to address the regularized NMF problem. Subsequently, we implement further applications in topic modeling and document clustering to verify the effectiveness of our newly proposed regularized NMF model and algorithms in word embedding and document embedding scenarios.

## Related background

In this section, we introduce some background information about the construction of the term-document matrix and the word-context matrix, which are used in our proposed NMF model.

In line with LSA, the standard NMF begins with the construction of the term-document matrix [8]. As its name suggests, this matrix’s rows represent term vectors and its columns represent document vectors. Typically, what constitutes a term or a document in a given document corpus can vary depending on the application. Generally, a document collection consisting of *m* terms and *n* documents is represented as an *m* × *n* term-document matrix *A*. For common document collections such as newspapers and magazines, the number of terms is usually much greater than the number of documents (*m* ≫ *n*); however, there are also reversed situations (*n* ≫ *m*), particularly for documents sourced from webpages. Each element, *a*_*ij*_, of matrix *A* represents the frequency of term *i* in document *j*. Typically, most of these elements are zero, making matrix *A* generally quite sparse. Moreover, the element *a*_*ij*_ necessitates an appropriate weighting scheme that depends on the specific characteristics of the document collection. More precisely, the weighted version of *a*_*ij*_ can be defined as

$$\begin{array}{c} a_{ij}=l_{ij}\cdot g_{i}, \end{array}$$ (1)

where *l*_*ij*_ denotes a local weight for term *i* in document *j*, and *g*_*i*_ denotes a global weight for term *i* across the document collection. The typical choice for the local weight *l*_*ij*_ is the standard term frequency, denoted by *f*_*ij*_, while other options also exist, such as binary frequency (1 if the term is present in the document and 0 otherwise), and log frequency defined by log(*f*_*ij*_ + 1). The options for the global weight *g*_*i*_ include idf, entropy, normalization, and others, all of which are designed to assign lower weights to high-frequency terms that appear in many documents and higher weights to low-frequency terms that appear in specific documents. Overall, the selection of a combination of local and global weights results in the weighted frequency *a*_*ij*_ used in the term-document matrix *A*; commonly used combinations include tf-idf, log-entropy, etc.

Having constructed the term-document matrix *A*, the NMF attempts to approximate it by factorizing it into two reduced-rank nonnegative matrices *W* and *H*, such that *A* ≈ *WH*. The row vectors of the matrix *W* and the column vectors of the matrix *H* are both situated within a *k*-dimensional vector space. They can be used as word embeddings and document embeddings respectively, and can subsequently be employed for applications such as clustering and classification. The approximation can be formulated mathematically as follows: given a nonnegative matrix $A\in R^{m\times n}$ and a positive integer *k* ≤ min{*m*, *n*}, find two nonnegative matrices $W\in R^{m\times k}$ and $H\in R^{k\times n}$ to minimize the cost function

$$\begin{array}{c} f\left(W,H\right)=\frac{1}{2}{\left\|A-WH\right\|}^{2}, \end{array}$$ (2)

where ‖ ⋅ ‖ denotes the Frobenius norm which calculates the square root of the sum of squares of all elements in the input matrix.

Before moving on to consider the word-context matrix, it is worth noting here that, in fact, there have been extensive alternate formulations of the NMF model since its initial introduction. For example, instead of using the Frobenius norm in (2), various other measures have been proposed to quantify the difference between *A* and *WH*. Among these measures, the Kullback-Leibler divergence and its variants are particularly noteworthy, while a multitude of alternative approaches to formulating the cost function still exist in the literature. However, as mentioned earlier, we will focus on the regularization approach in this paper.

To implement the regularization, the construction of a word-context matrix is still needed for our proposed model in addition to the term-document matrix. Informally speaking, a word-context matrix is a matrix representation for text data whose elements represent co-occurrences between words within a local context. To make it more explicit, we carry forward the basic idea of global embedding models like Word2vec and GloVe: the co-occurrence statistics *c*_*ii*′_ can be determined to indicate the number of times term *i*′ occurs in the context of term *i* (the context of a term could be a local word window as in Word2vec). As mentioned earlier, the SGNS (Skip-Gram with Negative Sampling) model within the Word2vec framework can be regarded as factorizing the PMI (Pointwise Mutual Information) matrix, shifted by the log of the number of negative samples *N*, which can further be approximated by a nonnegative matrix $S=\left(s_{ii^{\prime }}\right)\in R^{m\times m}$ as follows [18]:

$$\begin{array}{c} s_{ii^{\prime }}=\text{max}\{\text{log}\;\frac{c_{ii^{\prime }}c_{\cdot \cdot }}{c_{i\cdot }c_{\cdot i^{\prime }}}-\text{log}\;N,\;0\}, \end{array}$$ (3)

where *c*_⋅⋅_ = ∑_*ii*′_ *c*_*ii*′_, *c*_*i*⋅_ = ∑_*i*′_ *c*_*ii*′_ and *c*_⋅*i*′_ = ∑_*i*_*c*_*ii*′_. For the sake of clarity, the aforementioned matrix *S* is used as the word-context matrix for subsequent discussion. Given that *S* is typically an SPD matrix, we are now prepared to establish the aforementioned regularized NMF model.

## The proposed method

Building upon the cost function of NMF in (2), we propose a regularization scheme to simultaneously approximate the term-document matrix *A* and the word-context matrix *S* as follows:

$$\begin{array}{c} f_{\lambda }\left(W,H\right)=\frac{1}{2}{\left\|A-WH\right\|}^{2}+\frac{\lambda }{2}D\left(S,WW^{T}\right). \end{array}$$ (4)

Here, λ ≥ 0 is the regularization parameter, which can be predetermined or adjusted by cross-validation depending on the problem. The regularization term *D*(*S*, *WW*^*T*^) requires the utilization of a metric *D*(⋅, ⋅) to quantify the distance between the word-context matrix *S* and the approximating factorization *WW*^*T*^. The simplest choice for the metric *D*(⋅, ⋅) might be the squared Euclidean distance induced by the Frobenius norm, which has been investigated in the literature [24].

However, the Euclidean distance does not capitalize on the fact that *S* is an SPD matrix. Due to this, the Wasserstein metric on the SPD manifold—that is, the intrinsic curved space composed of SPD matrices—is utilized for regularization in place of the Euclidean distance. Originating from the background of optimal transportation, the Wasserstein metric has been extensively studied in the literature of statistics and machine learning [25, 26]. In particular, the geometric structure of the SPD manifold allows for an explicit formulation of the Wasserstein metric, which, in turn, enables the development of additional useful geometric concepts such as geodesics and Riemannian gradients [23].

To take advantage of the SPD manifold structure, the metric *D*(⋅, ⋅) in (4) is selected as the squared Wasserstein metric for later use in this paper; its explicit formulation is as follows [23]:

$$\begin{array}{c} D\left(A,B\right)=\text{tr}\left(A\right)+\text{tr}\left(B\right)-2\;\text{tr}[{\left(B^{1/2}AB^{1/2}\right)}^{1/2}]. \end{array}$$ (5)

Here, It is important to note that the matrix square root operation is needed in the calculation of the third term in (5).

With the metric *D*(⋅, ⋅) given as above, the proposed regularized NMF problem can be stated almost in the same way as before: given the nonnegative term-document matrix $A\in R^{m\times n}$ and word-context matrix *S*, along with a positive integer *k* ≤ min{*m*, *n*}, we need to find two nonnegative matrices $W\in R^{m\times k}$ and $H\in R^{k\times n}$ to minimize the cost function *f*_λ_(*W*, *H*) as given in (4). Once this regularized NMF problem is solved, the row vectors of matrix *W* and the column vectors of matrix *H* should be used as word embeddings and document embeddings, respectively, as before.

Before proceeding to demonstrate the numerical approaches to solving the regularized NMF problem, we briefly mention the numerical algorithms of the standard NMF model. These algorithms are typically implemented in iterative processes; hence, an initialization of *W* and *H* is usually performed at the start of the iteration. While initializing with random nonnegative values might be a simple practical option, numerous works have been devoted to alternative initialization approaches [27–30]. Returning to the general scheme of the iteration, although various specialized NMF iterative algorithms exist, they are typically categorized into three primary classes: multiplicative update algorithms, gradient descent algorithms, and alternating least squares algorithms. We now provide a brief review of these three classes.

The multiplicative update algorithms have been a useful baseline for comparison with other algorithms due to their easy-to-implement formulation [31]. The outline of the multiplicative update algorithm is summarized in Algorithm 1.

**Algorithm 1**: Multiplicative update algorithm for NMF with cost function (2)

 **Input**: $A\in R^{m\times n}$, *k* ≤ min{*m*, *n*}, maxiter, *ϵ* > 0,

 **Output**: $W\in R^{m\times k}$, $H\in R^{k\times n}$

1 Initialize *W* and *H* as random nonnegative matrices;

2 **for** *i* ← 1 **to** maxiter **do**

3  *H* ← *H*. * (*W*^*T*^*A*)./(*W*^*T*^*WH* + *ϵ*);

4  *W* ← *W*. * (*AH*^*T*^)./(*WHH*^*T*^ + *ϵ*);

5 **end**

Here, maxiter denotes the maximum iteration number, and *ϵ* is a small positive floating-point number used to avoid division by zero. The operations .* and ./ represent element-wise multiplication and element-wise division, respectively.

The gradient descent algorithms are among the most widely used classes for NMF. As its name suggests, a gradient descent algorithm takes each iteration step in the direction of the negative gradient, which is the direction of the steepest descent. The outline of the basic version of the gradient descent algorithm is summarized in Algorithm 2.

**Algorithm 2**: Gradient descent algorithm for NMF with cost function (2)

 **Input**: $A\in R^{m\times n}$, *k* ≤ min{*m*, *n*}, maxiter, *ϵ*_*W*_, *ϵ*_*H*_ > 0,

 **Output**: $W\in R^{m\times k}$, $H\in R^{k\times n}$

1 Initialize *W* and *H* as random nonnegative matrices;

2 **for** *i* ← 1 **to** maxiter **do**

3  $H\leftarrow H-\epsilon_{H}\frac{\partial f}{\partial H}$;

4  Set negative elements in *H* to 0;

5  $W\leftarrow W-\epsilon_{W}\frac{\partial f}{\partial W}$;

6  Set negative elements in *W* to 0;

7 **end**

Here, *ϵ*_*W*_ and *ϵ*_*H*_ are step size parameters; their values should be carefully chosen to achieve efficient convergence [32]. To ensure the nonnegativity of the updated matrices *H* and *W* in each iteration, a common practice is to set all negative elements to 0 before the next iteration in this and the following algorithms. The partial derivatives of the cost function (2) are explicitly calculated as follows:

$$\begin{array}{c} \begin{array}{cc} \frac{\partial f}{\partial H} & =-W^{T}\left(A-WH\right),\\ \frac{\partial f}{\partial W} & =-\left(A-WH\right)H^{T}. \end{array} \end{array}$$ (6)

The class of alternating least squares algorithms derives its name from the fact that these algorithms are implemented by using least squares steps in an alternating fashion. Its usage for NMF dates back to an early time, similar to the other two classes of algorithms [33]. The basic idea is to solve one of the matrices, *H* or *W*, with the other matrix fixed, which amounts to a least squares problem in each step. The outline of an elementary alternating least squares algorithm is summarized in Algorithm 3.

**Algorithm 3**: Alternating least squares algorithm for NMF with cost function (2)

 **Input**: $A\in R^{m\times n}$, *k* ≤ min{*m*, *n*}, maxiter

 **Output**: $W\in R^{m\times k}$, $H\in R^{k\times n}$

1 Initialize *W* as random nonnegative matrix;

2 **for** *i* ← 1 **to** maxiter **do**

3  *H* ← (*W*^*T*^*W*)^−1^*W*^*T*^*A*;

4  Set negative elements in *H* to 0;

5  *W* ← *AH*^*T*^(*HH*^*T*^)^−1^;

6  Set negative elements in *W* to 0;

7 **end**

In what follows, we propose modified algorithms for these three classes to accommodate our regularized NMF problem. It is appropriate to begin with an introduction to the gradient computation for the regularized term in the cost function (4).

### Gradient computation for regularized term

It is shown that the SPD manifold, equipped with the Wasserstein metric given by (5), admits explicit expressions for the geodesics and the partial derivatives of the Wasserstein metric [23]. The geodesics are curves of locally minimal distance, and their expression is as follows:

$$\begin{array}{c} {\text{Exp}}_{P}\left(tV\right)=\left(I+tX\right)P\left(I+tX\right). \end{array}$$ (7)

Here, Exp_*P*_(*tV*) denotes the geodesic starting from matrix *P* with an initial velocity *V*. Matrix *P* is an SPD matrix, and *V* is given by *XP* + *PX*, where *X* is symmetric. Furthermore, the partial derivatives of the squared Wasserstein metric *D*(*A*, *B*) with respect to *A* are given by

$$\begin{array}{c} \frac{\partial D}{\partial A}=I-B^{1/2}{\left(B^{1/2}AB^{1/2}\right)}^{-1/2}B^{1/2}. \end{array}$$ (8)

We will denote the right-hand side of (8) by *δ*(*A*, *B*) henceforth. Given that *D*(*A*, *B*) is symmetric, it is important to note that

$$\begin{array}{c} \frac{\partial D}{\partial B}=\delta \left(B,A\right). \end{array}$$ (9)

Now we focus on the gradient computation for the regularized term in (4), which will be denoted by

$$\begin{array}{c} \varphi \left(W\right)≔\frac{1}{2}D\left(S,WW^{T}\right). \end{array}$$ (10)

It turns out that the Riemannian gradient, i.e., the steepest direction along a geodesic given by (7), of *φ* with respect to *WW*^*T*^ is determined by *δ*(*WW*^*T*^, *S*), which leads to the expression of the gradient (partial derivatives) of *φ* with respect to *W*. As this result will be used in subsequent discussions, we state the following fundamental result and provide its proof.

**Proposition 1**. *The gradient of the regularized term φ in* (10) *with respect to W is given by*

$$\begin{array}{c} \frac{\partial \varphi }{\partial W}=\delta \left(WW^{T},S\right)W. \end{array}$$ (11)

*Proof*. We try to obtain a matrix *G* such that

$$\begin{array}{c} \frac{\text{d}}{\text{d}t}{|}_{t=0}\varphi \left(W+t\Delta W\right)=\text{tr}\left(\Delta W^{T}G\right), \end{array}$$ (12)

then it could be concluded that $\frac{\partial \varphi }{\partial W}=G$. We perform the following calculation:

$$\begin{array}{c} \begin{array}{cc} \frac{\text{d}}{\text{d}t}{|}_{t=0}\varphi \left(W+t\Delta W\right) & =\frac{1}{2}\frac{\text{d}}{\text{d}t}{|}_{t=0}D\left(S,\left(W+t\Delta W\right){\left(W+t\Delta W\right)}^{T}\right)\\ & =\frac{1}{2}\text{tr}\left[\delta {\left(WW^{T},S\right)}^{T}\left(\Delta WW^{T}+W\Delta W^{T}\right)\right]\\ & =\frac{1}{2}\text{tr}\left[W^{T}\delta {\left(WW^{T},S\right)}^{T}\Delta W\right]+\frac{1}{2}\text{tr}\left[\Delta W^{T}\delta {\left(WW^{T},S\right)}^{T}W\right]\\ & =\frac{1}{2}\text{tr}\left[\Delta W^{T}\delta \left(WW^{T},S\right)W\right]+\frac{1}{2}\text{tr}\left[\Delta W^{T}\delta \left(WW^{T},S\right)W\right]\\ & =\text{tr}[\Delta W^{T}\delta \left(WW^{T},S\right)W]. \end{array} \end{array}$$ (13)

Note that, the *δ*(*WW*^*T*^, *S*) is derived from (9), and it is a symmetric matrix. Hence, the desired conclusion follows.

Unfortunately, the theoretical computation of *δ*(*WW*^*T*^, *S*) in (11) is problematic in practice, since it involves the inversion of a reduced-rank matrix. To address this issue, we propose to replace the ordinary matrix inverse with the Moore-Penrose pseudoinverse for the computation of the gradient. The explicit result is summarized as follows.

**Proposition 2**. *Suppose the rank-k matrix S*^1/2^*W admits an SVD given by*

$$\begin{array}{c} S^{1/2}W=U\Sigma V^{T}, \end{array}$$ (14)

*where* $U\in R^{m\times k},V\in R^{k\times k}$ *are orthogonal*, Σ *is diagonal with diagonal elements being the singular values σ*_1_, ⋯, *σ*_*k*_. *Then, with the ordinary inverse being replaced with the Moore-Penrose pseudoinverse, the computation of the gradient of the regularized term φ with respect to W is given by*

$$\begin{array}{c} \frac{\partial \varphi }{\partial W}=W-S^{1/2}UV^{T}. \end{array}$$ (15)

*Proof*. We first note that

$$\begin{array}{c} \begin{array}{cc} {\left(S^{1/2}WW^{T}S^{1/2}\right)}^{1/2} & ={\left[\left(U\Sigma V^{T}\right)\left(V\Sigma U^{T}\right)\right]}^{1/2}\\ & ={\left(U\Sigma^{2}U^{T}\right)}^{1/2}\\ & =U\Sigma U^{T}. \end{array} \end{array}$$ (16)

Consequently, the Moore-Penrose pseudoinverse of (*S*^1/2^*WW*^*T*^*S*^1/2^)^1/2^ is equal to *U*Σ^−1^*U*^*T*^. Furthermore, we have

$$\begin{array}{c} \delta \left(WW^{T},S\right)=I-S^{1/2}\left(U\Sigma^{-1}U^{T}\right)S^{1/2}, \end{array}$$ (17)

By (11), the desired result is obtained as

$$\begin{array}{c} \begin{array}{cc} \frac{\partial \varphi }{\partial W} & =W-S^{1/2}\left(U\Sigma^{-1}U^{T}\right)S^{1/2}W\\ & =W-S^{1/2}U\Sigma^{-1}U^{T}U\Sigma V^{T}\\ & =W-S^{1/2}U\Sigma^{-1}\Sigma V^{T}\\ & =W-S^{1/2}UV^{T}. \end{array} \end{array}$$ (18)

Note that, the third equality makes use of the fact that the orthogonality of *U* implies that *U*^*T*^*U* = *I*.

The above results can be immediately applied to the computation of the partial derivatives of the cost function (4) as follows:

$$\begin{array}{c} \begin{array}{cc} \frac{\partial f_{\lambda }}{\partial H} & =\frac{\partial f}{\partial H}=-W^{T}\left(A-WH\right),\\ \frac{\partial f_{\lambda }}{\partial W} & =\frac{\partial f}{\partial W}+\lambda \;\frac{\partial \varphi }{\partial W}=-\left(A-WH\right)H^{T}+\lambda \left(W-S^{1/2}UV^{T}\right). \end{array} \end{array}$$ (19)

### Fundamental algorithms for regularized NMF

The proposed regularized NMF problem can be formulated as a minimization problem with inequality constraints:

$$\begin{array}{c} \underset{W,H}{\text{min}}\;f_{\lambda }\left(W,H\right)\;\text{subject}\;\text{to}\;W,H\geq 0. \end{array}$$ (20)

Let $\alpha \in R^{m\times k},\;\beta \in R^{k\times n}$ be the Lagrange multipliers for the constraints, then the Lagrangian function for the problem (20) is given by

$$\begin{array}{c} L\left(W,H,\alpha ,\beta \right)=f_{\lambda }\left(W,H\right)-\text{tr}\left(\alpha^{T}W\right)-\text{tr}\left(\beta^{T}H\right). \end{array}$$ (21)

Consequently, the Karush-Kuhn-Tucker conditions are given by

$$\begin{array}{c} \begin{array}{cc} & \alpha ,\beta \geq 0,\\ & \frac{\partial f_{\lambda }}{\partial W}=\alpha ,\;\frac{\partial f_{\lambda }}{\partial H}=\beta ,\\ & \alpha .*W=0,\;\beta .*H=0. \end{array} \end{array}$$ (22)

Combining (22) with (19), the following equations are obtained:

$$\begin{array}{c} \begin{array}{cc} & \left(W^{T}WH\right).*H=\left(W^{T}A\right).*H,\\ & \left[W\left(HH^{T}+\lambda I\right)\right].*W=\left(AH^{T}+\lambda S^{1/2}UV^{T}\right).*W. \end{array} \end{array}$$ (23)

Based on (23), a modified version of the multiplicative update algorithm for the regularized NMF model is derived. Compared with the original algorithm for NMF, the major difference with the modified algorithm lies in the fact that the update formula for matrix *W* now includes additional terms involving the regularization parameter λ and the matrix *S*. Notably, the implementation of SVD is required before each update for *W*. The outline of this algorithm is summarized in Algorithm 4.

**Algorithm 4**: Multiplicative update algorithm for regularized NMF with cost function (4)

 **Input**: $A\in R^{m\times n}$, $S\in R^{m\times m}$, *k* ≤ min{*m*, *n*}, λ ≥ 0, maxiter, *ϵ* > 0,

 **Output**: $W\in R^{m\times k}$, $H\in R^{k\times n}$

1 Initialize *W* and *H* as random nonnegative matrices;

2 **for** *i* ← 1 **to** maxiter **do**

3  *H* ← *H*. * (*W*^*T*^*A*)./(*W*^*T*^*WH* + *ϵ*);

4  Implement the SVD: *S*^1/2^*W* = *U*Σ*V*^*T*^ (the previously updated *W* is used);

5  *W* ← *W*. * (*AH*^*T*^ + λ*S*^1/2^*UV*^*T*^)./[*W*(*HH*^*T*^ + λ*I*) + *ϵ*];

6 **end**

The proposed gradient descent algorithm for regularized NMF is basically the same as before, except that the partial derivatives of the cost function *f* is replaced with those of the cost function *f*_λ_ which are determined by (19). Note that the difference between (6) and (19) lies in that $\frac{\partial f_{\lambda }}{\partial W}$ includes an additional correction term involving the regularization parameter λ and the matrix *S*. The implementation of SVD is also required for the computation of $\frac{\partial f_{\lambda }}{\partial W}$ in each iteration step. In addition, the Karush-Kuhn-Tucker conditions (22) can be used as the convergence criterion for the iteration of the modified gradient descent algorithm. The outline of this algorithm is summarized in Algorithm 5.

**Algorithm 5**: Gradient descent algorithm for regularized NMF with cost function (4)

 **Input**: $A\in R^{m\times n}$, $S\in R^{m\times m}$, *k* ≤ min{*m*, *n*}, λ ≥ 0, maxiter, *ϵ*_*W*_, *ϵ*_*H*_ > 0,

 **Output**: $W\in R^{m\times k}$, $H\in R^{k\times n}$

1 Initialize *W* and *H* as random nonnegative matrices;

2 **for** *i* ← 1 **to** maxiter **do**

3  Calculate $\frac{\partial f_{\lambda }}{\partial H}$ and $\frac{\partial f_{\lambda }}{\partial W}$ by (19);

4  $H\leftarrow H-\epsilon_{H}\frac{\partial f_{\lambda }}{\partial H}$;

5  Set negative elements in *H* to 0;

6  $W\leftarrow W-\epsilon_{W}\frac{\partial f_{\lambda }}{\partial W}$;

7  Set negative elements in *W* to 0;

8 **end**

The proposed alternating least squares algorithm for regularized NMF is derived by setting the partial derivatives $\frac{\partial f_{\lambda }}{\partial H}$ and $\frac{\partial f_{\lambda }}{\partial W}$ in (19) to 0, and then solving for *H* and *W* alternatively. The significant change in the modified alternating least squares algorithm is that, in each iteration, *W* is updated with additional terms that involve the regularization parameter λ and the matrix *S*. The outline of this algorithm is summarized in Algorithm 6.

**Algorithm 6**: Alternating least squares algorithm for regularized NMF with cost function (4)

 **Input**: $A\in R^{m\times n}$, $S\in R^{m\times m}$, *k* ≤ min{*m*, *n*}, λ ≥ 0, maxiter

 **Output**: $W\in R^{m\times k}$, $H\in R^{k\times n}$

1 Initialize *W* as random nonnegative matrix;

2 **for** *i* ← 1 **to** maxiter **do**

3  *H* ← (*W*^*T*^*W*)^−1^*W*^*T*^*A*;

4  Set negative elements in *H* to 0;

5  Implement the SVD: *S*^1/2^*W* = *U*Σ*V*^*T*^ (the previously updated *W* is used);

6  *W* ← (*AH*^*T*^ + λ*S*^1/2^*UV*^*T*^)(*HH*^*T*^ + λ*I*)^−1^;

7  Set negative elements in *W* to 0;

8 **end**

At last, we should note that although the maximum number of iterations was used as the stopping criterion in all these algorithms, as demonstrated above, it is challenging to set a fixed number of iterations, as the specific choice often depends on the problem at hand. In light of this issue, alternative criteria might be suggested for practical use; however, we will not explore this further in this paper. Instead, we will focus on analyzing specific experiments for text embedding tasks. We shall find that the additional computational burden imposed by SVD is worthwhile, as it effectively addresses the aforementioned issue of semantic loss, thereby validating the effectiveness of the proposed algorithms.

## Experimental results and discussion

This section is committed to presenting experiments designed to address threefold purposes. Firstly, to verify the effectiveness of the three proposed algorithms for our Wasserstein-regularized NMF model, we compare the convergence of these algorithms when implemented on a reduced version of the benchmark 20NG dataset [34]. Secondly, using the same reduced 20NG dataset, we perform topic modeling separately with NMF and regularized NMF, thereby validating from the perspective of word embedding that the regularized NMF can address the issue of semantic loss. Finally, to further confirm the resolution of this issue from the perspective of document embedding, we will conduct document clustering on three benchmark datasets to compare the performance of our proposed model with that of several other commonly used models.

All the experiments in this section are implemented within the Python environment. The 20NG dataset can be accessed from the scikit-learn library, while the other two datasets (IMDB and BBC News) used in our study are included as S1 Data. We primarily used the TfidfVectorizer transformer in scikit-learn to obtain the tf-idf representation of the term-document matrix. Additionally, some commonly used text preprocessing techniques were employed by properly setting the optional parameters of TfidfVectorizer. Specifically, the built-in list of English stopwords in scikit-learn was used throughout our experiments. Furthermore, high-frequency and low-frequency terms were discarded by setting the max_df and min_df options of TfidfVectorizer to obtain an appropriate number of features. No other advanced text normalization techniques were pursued in this study.

### Convergence comparison of regularized NMF algorithms

We now compare the performances of the multiplicative update (MU), gradient descent (GD), and alternating least squares (ALS) algorithms for the regularized NMF model. The term-document matrix *A* and the word-context matrix are constructed from the aforementioned reduced 20NG dataset. The full 20NG dataset contains a large number of newsgroup posts on 20 topics. For the sake of convenience in our presentation, the reduced dataset is obtained from the full 20NG dataset by selecting four topics with specified category names.

**Fig 1:**
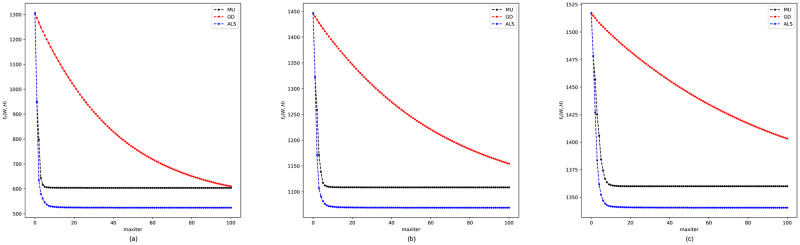
Convergence curves of MU, GD, and ALS algorithms for the regularized NMF. The regularization parameter λ is chosen among three different values: (**a**) regularization with λ = 0.5, (**b**) regularization with λ = 1, (**c**) regularization with λ = 2.

Throughout our experiments, the term-document matrix *A* is obtained by tf-idf representation as described in (1), and the word-context matrix is constructed using (3) with utilization of a 5-word window to capture the context of a term. Based on these constructions, our proposed regularized NMF model is established and implemented with the three proposed algorithms. To investigate the influence of different values of the regularization parameter λ, we selected λ = 0.5, λ = 1 and λ = 2 for comparison. The convergence curves for the three algorithms under these conditions are presented in Fig 1.

As can be seen from Fig 1, both the MU and ALS algorithms converge rapidly, requiring only a few iteration steps in all three scenarios. However, the MU algorithm seems to attain only a local minimum, whereas the ALS algorithm yields a better final result. With a relatively low learning rate of 0.01 set, the GD algorithm exhibits a slow but seemingly stable convergence, which is apparently influenced by the value of λ. Specifically, the smallest regularization value of λ = 0.5 demonstrates the best convergence in this experiment.

As mentioned earlier, it should be emphasized that the performance of these algorithms may depend on specific choices for initialization, step size, number of iterations, and so on. Furthermore, particular acceleration techniques for these algorithms may be proposed. For our purposes, we are satisfied with the fact that these basic algorithms are sufficiently effective for our later applications. As suggested by Fig 1, the ALS algorithm will be utilized in subsequent experiments involving the implementation of the regularized NMF model.

### Word embedding for topic modelling

Topic modelling, which is a broad term describing the task of assigning each document to one or more topics, is one of the commonly used techniques in text mining. To achieve a reasonable distribution of topics, it is necessary to assign appropriate embedding representations to each term. While the standard NMF model serves as a popular scheme for word embedding tasks, it has been noted that there is a significant loss of semantic relationships between words. Therefore, the proposed regularized NMF model is expected to improve upon the ordinary NMF model. We verify this by implementing both models on the reduced 20NG dataset. Since the reduced 20NG dataset is obtained by specifically selecting 4 topics from the 20 topics in the full dataset, the number of extracted features is also set to *k* = 4 in the setting of both models. Thus, after performing NMF and regularized NMF, each row vector of the term factor matrix *W* gives rise to a 4-dimensional representation of each term, which subsequently provides the word embedding for topic modelling.

**Fig 2:**
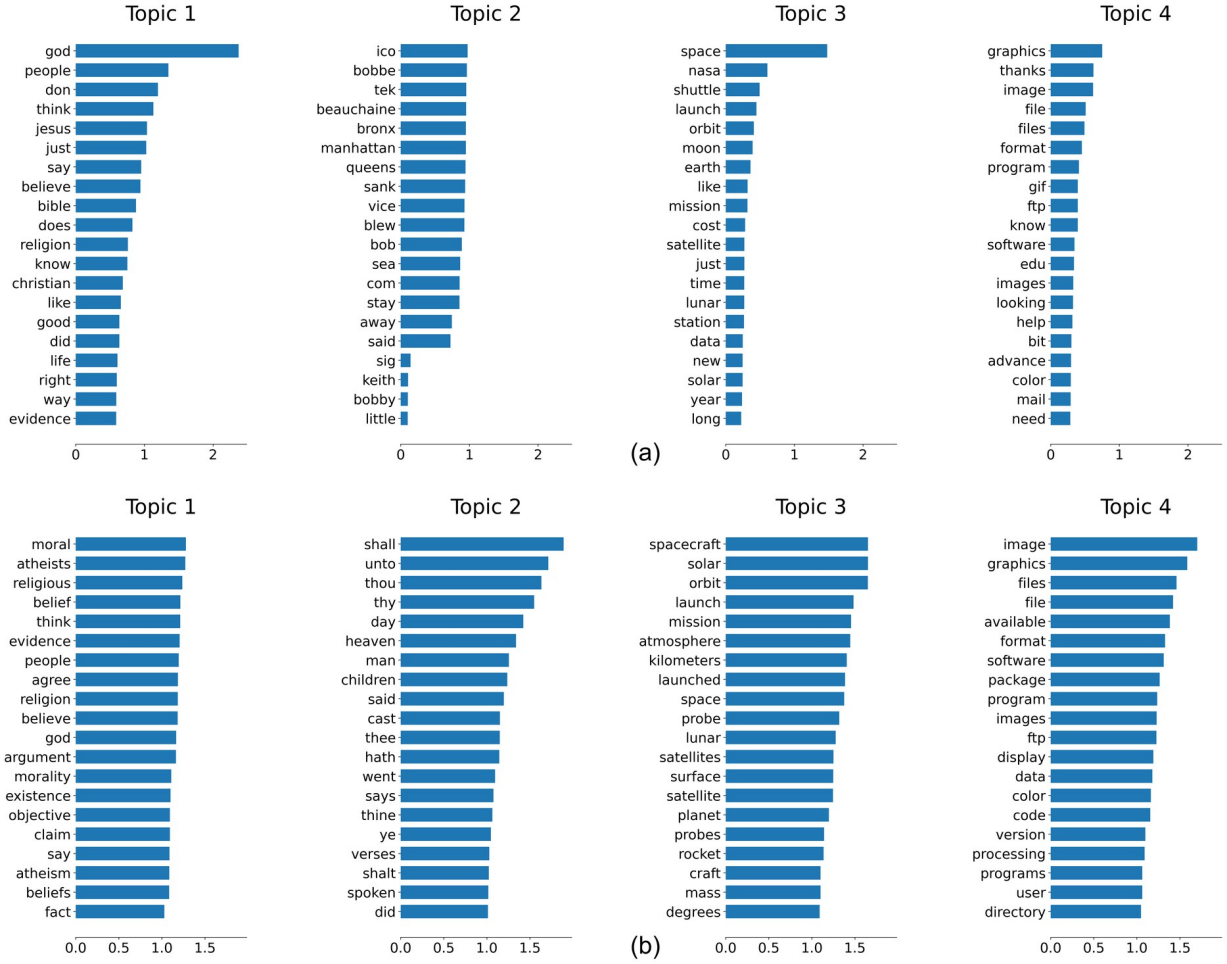
Barplot of top 20 words for each topic. Comparison is shown between (**a**) the NMF model and (**b**) the regularized NMF model.

The top 20 words for each topic, extracted by utilization of the factor matrix *W* from NMF and regularized NMF, are illustrated in Fig 2. The four topics are separately represented by the four columns of *W*; hence, in each column, the top 20 largest elements correspond to the top 20 words for each topic. These four selected topics in the reduced 20NG dataset have the following category names: “alt.atheism”, “talk.religion.misc”, “sci.space”, and “comp.graphics”. As shown in Fig 2, both models are successful in selecting topic-specific words. Upon closer inspection, it can be observed that the NMF model has introduced a few irrelevant words to the topics (e.g., the second word “thanks” in topic 4), whereas the words selected by the Regularized NMF model are highly relevant to the topics and exhibit a smoother distribution. Consequently, it is qualitatively verified that the regularized NMF model incorporates a significant amount of semantic information compared to the standard NMF model.

**Fig 3:**
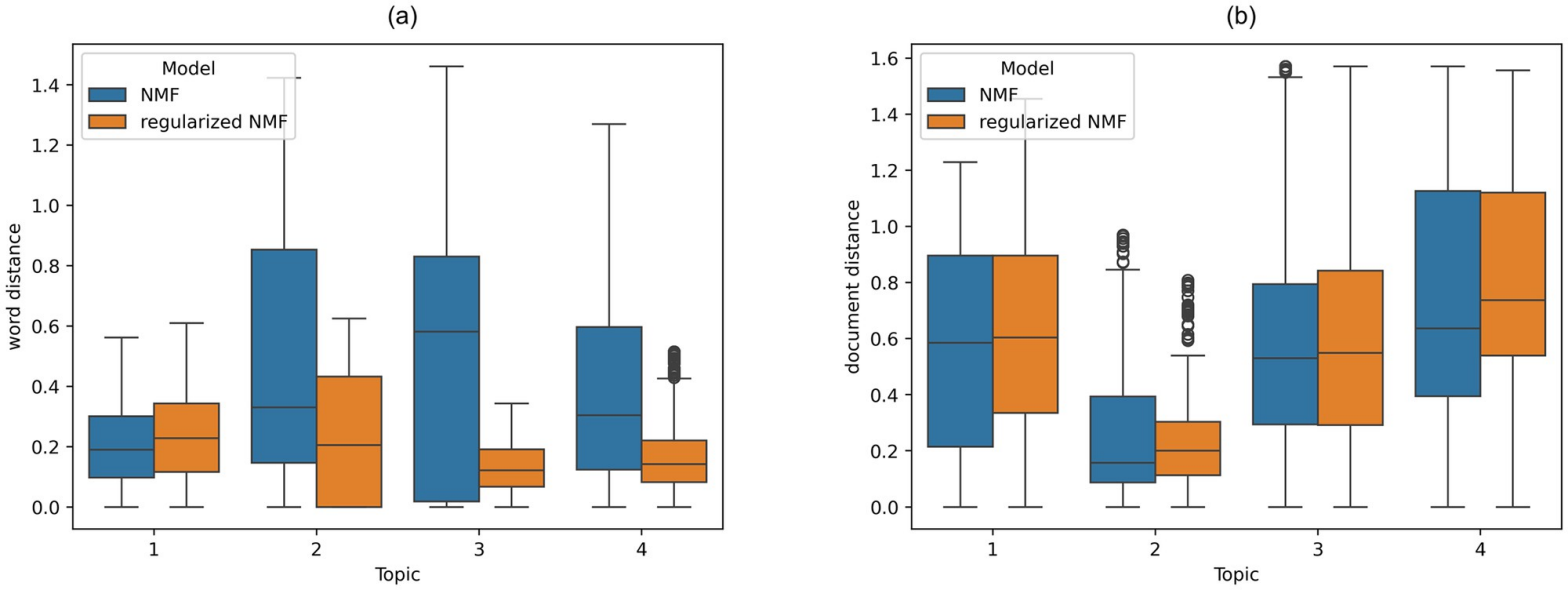
The distribution of the distances measured by vector angles between vector representations. The representations are intended for (**a**) top 30 words and (**b**) 20 selected documents within each topic, with comparison for NMF vs. regularized NMF.

For further quantitative verification, the vector angle, calculated as the inverse cosine of the cosine similarity, is utilized to measure the distance between embedding vectors. Formally, the vector angle between vectors *X* and *Y* is given by

$$\begin{array}{c} \theta \left(X,Y\right)=\text{arccos}(\frac{\left\langle X,Y\right\rangle }{\left\|X\|\|Y\right\|}), \end{array}$$ (24)

where 〈⋅, ⋅〉 denotes the inner product function, and ‖⋅‖ denotes the vector length function. The distribution of the vector angles between the word embedding vectors (i.e. the row vectors of *W*) within each topic is demonstrated in Fig 3(a). It is apparent that the top words within each topic tend to admit smaller distances under regularized NMF than under NMF. This further confirms that regularized NMF provides more similar word embeddings than NMF for capturing semantics, by assigning the representations of words that pertain to the same topic closer to each other in the latent semantic space.

The distribution of vector angle distances between the document embedding vectors (i.e., the column vectors of the document factor matrix *H*) within each topic is also illustrated in Fig 3(b) for reference. Although the differences between the two models are not significant in this instance, the effectiveness of regularized NMF for document embedding will be further validated through subsequent experiments focused on document clustering.

### Document embedding for clustering

To evaluate the document embedding obtained from the document factor matrix *H* in our regularized NMF model, we compare it with four popular models commonly used for document clustering: K-means with the tf-idf representation (TF-IDF) [35], LSA based on SVD (LSA-SVD) [36], NMF with Frobenius norm (NMF-Fro) [9], and NMF with Kullback-Leibler divergence (NMF-KL) [37].

Three popular datasets were selected for demonstrating the performance of our model under different scenarios: the full version of the 20NG dataset, the BBC News dataset [38], and the training subset of the IMDB dataset [39]. The basic characteristics of these datasets are summarized in Table 1. The size of the term-document matrix $A\in R^{m\times n}$ is determined by the number of documents *n* and the number of terms *m*. The number of true classes is taken as the reduced feature number *k* in all these NMF models, which is equal to the number of columns in *W* and the number of rows in *H*. The balance is defined for each dataset as the ratio of the smallest class size to the largest class size. The sparsity is defined as the percentage of nonzero elements in each of the term-document matrix *A* and the word-context matrix *S*.

**Table 1:** The basic characteristics of the datasets.

Dataset	No. of Documents	No. of Terms	No. of Classes	Balance	Sparsity of *A*	Sparsity of *S*
20NG	18846	8600	20	0.629	0.58%	4.17%
BBC News	2225	5604	5	0.755	0.85%	6.08%
IMDB	25000	8963	2	1	2.10%	3.89%

To quantify the quality of the clustering results, we shall employ three widely used evaluation metrics: the adjusted Rand index (ARI) [40], the adjusted mutual information (AMI) [41], and the V-measure [42]. These metrics are intended to measure the similarity between the predicted clusters and the true clusters. Higher values of these metrics indicate better clustering performance.

We have performed five runs with random initialization for each model to obtain average values and estimated errors of these metrics. These results are summarized in Table 2, where our proposed NMF model with Wasserstein regularization is denoted as NMF-WR. For the NMF-WR model, the regularization parameter λ was selected through random search from a predetermined range of possible values.

**Table 2:** The comparison of clustering evaluation metrics over different datasets.

Dataset	Metrics	TF-IDF	LSA-SVD	NMF-Fro	NMF-KL	NMF-WR
20NG	ARI	0.052 ± 0.029	0.204 ± 0.004	0.145 ± 0.000	0.188 ± 0.000	0.176 ± 0.000
	AMI	0.250 ± 0.087	0.337 ± 0.003	0.292 ± 0.001	0.296 ± 0.001	0.356 ± 0.001
	V-measure	0.252 ± 0.087	0.339 ± 0.003	0.282 ± 0.001	0.295 ± 0.000	0.344 ± 0.001
BBC News	ARI	0.762 ± 0.166	0.845 ± 0.000	0.704 ± 0.000	0.651 ± 0.000	0.803 ± 0.000
	AMI	0.783 ± 0.099	0.813 ± 0.000	0.714 ± 0.000	0.676 ± 0.000	0.835 ± 0.000
	V-measure	0.783 ± 0.098	0.813 ± 0.000	0.705 ± 0.000	0.676 ± 0.000	0.833 ± 0.000
IMDB	ARI	0.112 ± 0.003	0.145 ± 0.004	0.122 ± 0.000	0.144 ± 0.000	0.075 ± 0.000
	AMI	0.094 ± 0.003	0.108 ± 0.003	0.121 ± 0.000	0.109 ± 0.000	0.192 ± 0.000
	V-measure	0.094 ± 0.003	0.108 ± 0.003	0.114 ± 0.000	0.109 ± 0.000	0.148 ± 0.000

As can be seen from the results in Table 2, the performance of the NMF-WR model shows a noticeable superiority compared to other models, especially in terms of AMI and V-measure. In terms of ARI, the LSA-SVD model exhibits the best performance among all models, while the NMF-WR model is still comparable to it on the 20NG and BBC News datasets. The low ARI value of the NMF-WR model on the IMDB dataset is compensated by the improvement in the other two metrics, as all other models perform less well. There actually exists a well-known difficulty in dealing with the IMDB dataset, which is typically used for sentiment analysis [43].

In summary, our proposed NMF-WR model does exhibit an intrinsic ability to leverage semantic information for text embedding, which could be further utilized in applications such as topic modeling and document clustering. Moreover, efficient numerical algorithms for the NMF-WR model can be constructed and implemented, taking into account the three basic classes introduced above.

## Conclusion

In this paper, to address the issue of semantic loss in standard NMF models for text embedding, we propose a novel NMF scheme with a specially constructed regularization term. Existing regularization techniques for NMF primarily aim to shrink the resulting factor matrices, but the problem of semantic loss remains unresolved. Typically, the word-context matrix is constructed to incorporate semantic information for the pre-training of neural networks, yet its application in NMF is not widespread. Although semantic information is missing from the term-document matrix approximation in ordinary NMF, it can be captured through an appropriate word-context matrix approximation. Moreover, given that the word-context matrix is usually SPD, it is fitting to utilize the Wasserstein metric on the SPD manifold. Inspired by these insights, our proposed NMF model includes a regularization term that quantifies the approximation to the word-context matrix based on the Wasserstein metric. We then modify three basic algorithms from the standard NMF version to address this newly proposed Wasserstein-regularized NMF (NMF-WR) problem. The effectiveness of these algorithms is verified, and the NMF-WR model’s ability to resolve semantic loss for word embedding and document embedding is validated through experiments in topic modeling and document clustering on popular text datasets.

Overall, we have found that NMF-WR significantly outperforms other conventional NMF models in processing text embeddings. These findings contribute to the expansion of effective methods for text mining tasks within the field of NLP. Furthermore, the enhancements brought by NMF-WR suggest that assigning appropriate geometric structures, such as the Wasserstein SPD manifold structure used in our NMF-WR model, can greatly facilitate text analysis. Most notably, to our knowledge, our study is the first to integrate this geometric structure into text data analysis.

While our results provide evidence for the applicability of the NMF-WR model for text embedding and the effectiveness of the associated algorithms, some significant limitations remain. Although the basic algorithms proposed in this study are adequate for handling common datasets, a more refined approach to algorithm design and parameter selection could improve the efficiency of dealing with large sparse matrices derived from text data. Future research should, therefore, focus on addressing these challenges to further enhance the applicability of NMF-WR in a wider range of areas.

## Supporting information

S1 Data Data & code. (ZIP)
